# Chiral shape fluctuations and the origin of chirality in cholesteric phases of DNA origamis

**DOI:** 10.1126/sciadv.aaw8331

**Published:** 2020-07-29

**Authors:** Maxime M. C. Tortora, Garima Mishra, Domen Prešern, Jonathan P. K. Doye

**Affiliations:** 1 Physical and Theoretical Chemistry Laboratory, Department of Chemistry, University of Oxford, South Parks Road, Oxford OX1 3QZ, UK.; 2 Department of Physics, Indian Institute of Technology Kanpur, Kanpur 208016, India.

## Abstract

Lyotropic cholesteric liquid crystal phases are ubiquitously observed in biological and synthetic polymer solutions, characterized by a complex interplay between thermal fluctuations and entropic and enthalpic forces. The elucidation of the link between microscopic features and macroscopic chiral structure, and of the relative roles of these competing contributions on phase organization, remains a topical issue. Here, we provide theoretical evidence of a previously unidentified mechanism of chirality amplification in lyotropic liquid crystals, whereby phase chirality is governed by fluctuation-stabilized helical deformations in the conformations of their constituent molecules. Our results compare favorably to recent experimental studies of DNA origami assemblies and demonstrate the influence of intramolecular mechanics on chiral supramolecular order, with potential implications for a broad class of experimentally relevant colloidal systems.

## Teaser

Intramolecular mechanics govern supramolecular self-assembly in chiral liquid crystal phases of DNA origami filaments.

## INTRODUCTION

Linking the microscopic features of molecular building blocks to the material properties of their self-assembled macroscopic phases constitutes one of the overarching goals of modern colloidal science. The fascinating ability of colloidal systems to spontaneously form intricate, organized structures in the absence of external human intervention has inspired a considerable body of work over the past decades, fostered by rapid experimental progress in the synthesis of micro- and nanosized particles with complex shapes and tunable interactions (*1*). Colloidal self-assembly has thus emerged as a most promising route toward the bottom-up fabrication of functional nanomaterials, with potential applications spanning the fields of catalysis, energy harvesting, and drug delivery (*2*, *3*). However, the hierarchical nature of the colloidal assembly process—which involves the gradual propagation of orientational and/or positional order from molecular to macroscopic level—is generally challenging to rationalize and control, owing to both the diversity of physicochemical forces at play and the wide difference in length scales between elementary building blocks and supermolecular structures (*4*).

In this context, the question of the role of molecular chirality on colloidal organization has proven to be of singularly long-standing interest, from the point of view of both practical applications and fundamental research. The control of the chirality of a macroscopic material, along with the elucidation of its microscopic bases, has far-reaching implications ranging from pharmaceutical synthesis and photonics engineering (*5*, *6*) to the understanding of the origins of biological homochirality (*7*). In particular, the self-assembly of chiral molecular units into helical superstructures underlies the formation of the basic molecules of life—from the double-helical ordering of nucleotides in DNA to the α-helical arrangement of amino acids in protein secondary structures—and governs their remarkable ability to further organize into higher-order helical assemblies, such as helix-bundle proteins and protein-DNA complexes, which are essential to vital biological functions (*8*).

In colloidal systems, the most frequent manifestation of this so-called chirality amplification process lies in the lyotropic cholesteric liquid crystal (LChLC) phase, observed in solutions of many common chiral (bio)polymers in both in vivo and in vitro environments. The macroscopic breaking of mirror symmetry in LChLCs arises from the periodic rotation of the direction of local molecular alignment about a fixed normal axis as one passes through the sample, and may be fully quantified by the spatial period of this helical arrangement—termed as the cholesteric pitch. A remarkable feature of cholesterics, whose original discovery in 1888 is generally hailed as the birth of liquid crystal (LC) science (*9*), is the exquisite sensitivity of their pitch to subtle changes in the assembly conditions and chemical structure of their constituent particles. This delicate dependence has been studied in considerable detail in a variety of model systems, ranging from DNA duplex (*10*) and filamentous virus suspensions (*11*) to biologically relevant collagen assemblies (*12*), and forms the basis of an impressive array of potential applications in such diverse fields as cryptography, smart textiles, and physicochemical sensors (*13*).

Despite notable recent advances (*14*), the singular complexity and heterogeneity of the reported experimental phase behaviors have so far largely eluded attempts to resolve their microscopic underpinnings. While theoretical studies of simple particle models have uncovered a few general features of cholesteric organization, such as the nontrivial link between molecular and phase chirality (*15*–*17*), these investigations have been hindered by the strongly multiscale nature of the problem, as the cholesteric pitch of most common LChLCs usually lies in the micro- to millimeter range—several orders of magnitude larger than the typical molecular dimensions—which renders direct atomistic simulations largely impractical. Conversely, on the experimental side, the systematic analysis of the relationship between molecular structure and phase organization has been limited by the difficulties involved in the scalable fabrication of colloidal particles with addressable chirality. The establishment of a quantitative connection between molecular chirality and macroscopic helicity in LChLCs thus generally remains a major challenge of soft condensed matter physics, with broad consequences for their rational applications as bio-inspired multifunctional materials (*18*, *19*) and for our fundamental understanding of the ubiquitous occurrences of LChLC order in living matter (*20*).

Substantial progress in this direction has been recently achieved by exploiting the synergy between colloidal science and DNA origami technology, through which the LChLC organization of self-assembled origami filaments demonstrated the possibility to tune the micrometer-scale pitch of the bulk phase via the direct control of single-particle structure at the nanometer level (*21*). Through the conjunction of a well-established coarse-grained model of DNA with a classical molecular field theory of LChLCs, we here present a rigorous theoretical analysis of these experimental developments by assessing the detailed influence of particle mechanical properties and thermodynamic state on their ordering behavior, without the use of any adjustable parameters. These developments further enable us to demonstrate the importance of intramolecular fluctuations on chiral supramolecular organization and thus provide the first tangible evidence of a long-postulated, general mechanism of chirality amplification in biopolymer solutions (*11*). Each origami is comprised of a 7560-nucleotide scaffold strand, colored in yellow, and of ~200 shorter staple strands, colored independently for each origami variant, whose designed binding locations determine the filament ground state structure.

## RESULTS

### LChLC assembly of ground-state origamis

**Fig. 1:**
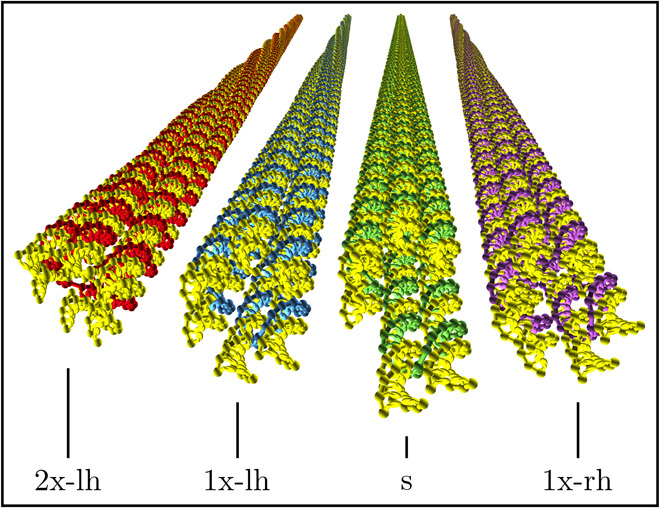
Ground-state origami conformations. Each origami is comprised of a 7,560-nucleotide scaffold strand, colored in yellow, and of ~200 shorter staple strands, colored independently for each origami variant, whose designed binding locations determine the filament ground-state structure. The equilibrium axial twist of the conformations is obtained by elastic energy minimization using a continuum DNA model (*23*). The nucleotide-level depiction corresponds to the finer-grained representation of the oxDNA model (*28*), as used in all mechanical calculations throughout the paper.

We consider monodisperse DNA bundles composed of six double helices crossed-linked in a tight hexagonal arrangement. These self-assembled filaments may be folded into shapes of programmable twist and curvature through targeted deletions and insertions of base pairs (bp) along each bundle (*22*). Following (*21*), we here focus on four variants of the filaments comprising 15,224 to 15,240 nucleotides, with experimentally determined contour lengths (*l*_c_) of 420 nm and bundle diameters (σ) of 6 nm. A continuum finite-element model based on an elastic rod description of DNA (*23*) predicts the respective ground states of the different designs to bear negligible (s), 360^∘^ right-handed (1x-rh), 360^∘^ left-handed (1x-lh), and 720^∘^ left-handed (2x-lh) twist about the filament long axis, with negligible net curvature (Fig. 1) (*21*).

As a first approximation, we neglect the conformational fluctuations of DNA origamis in solution and assess the cholesteric arrangement of their respective ground states. To that end, we make use of an efficient and accurate numerical implementation of the Onsager theory extended to the treatment of cholesteric order (*24*), which has been extensively discussed elsewhere (see Materials and Methods) (*25*, *26*). In this framework, the reliable investigation of their LChLC assembly requires the input of a mechanical model capable of resolving the local double-helical arrangement of nucleotides within each duplex (*27*). We thus use the oxDNA model (*28*), coarse-grained at the nucleotide level, to represent the origami microscopic structure and interaction potential (Fig. 1).

**Fig. 2:**
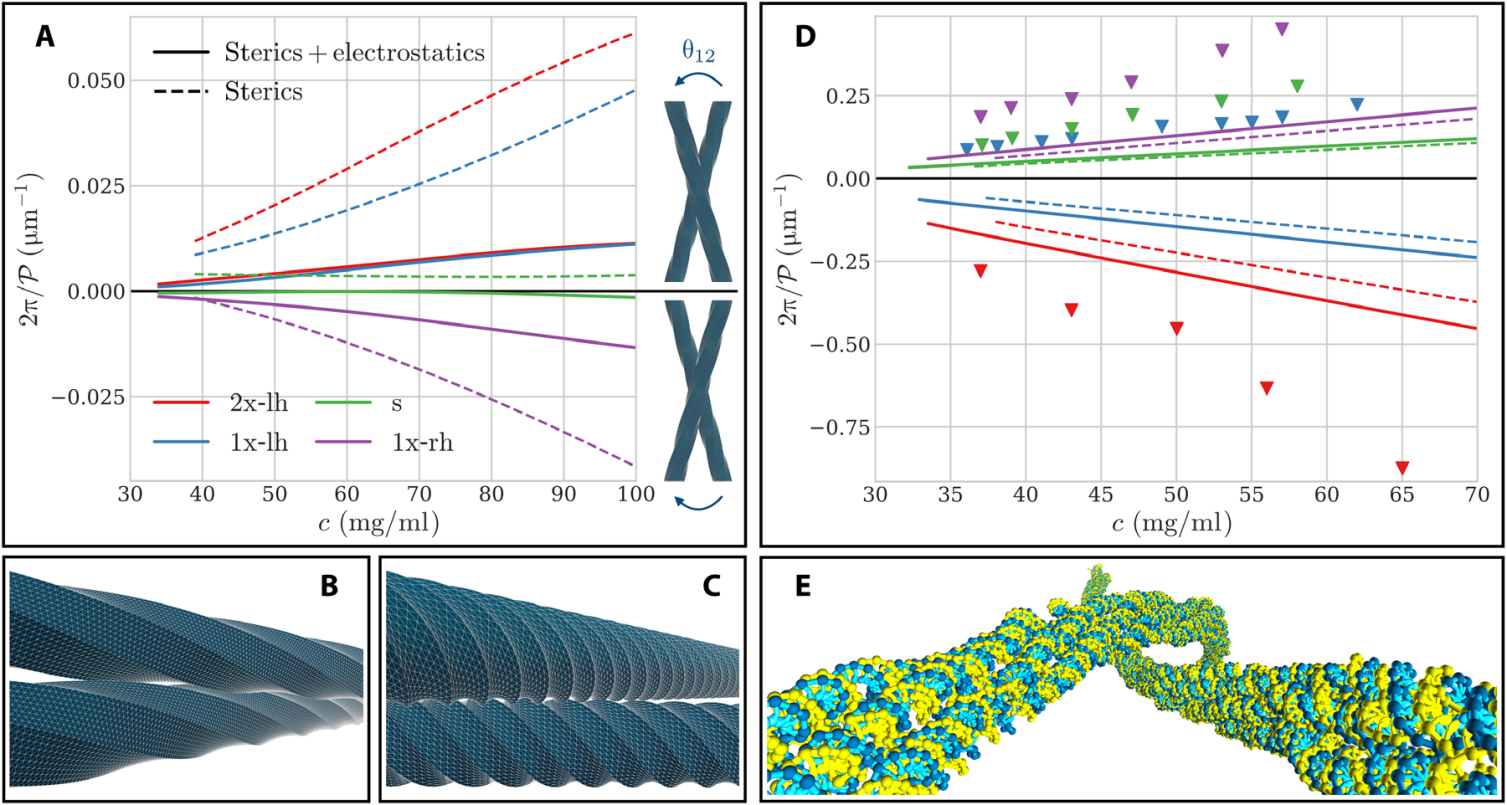
Cholesteric assembly of ground-state and thermalized origamis. (**A**) Inverse equilibrium cholesteric pitch (𝒫) as a function of particle concentration (*c*) for ground-state filament conformations. Dashed lines denote values obtained by assuming pure steric interactions and solid lines by accounting for both steric and Debye-Hückel repulsion. Positive (resp. negative) values of 𝒫 correspond to LChLC phases bearing right (resp. left) handedness, as illustrated in the right-hand panel. (**B**) Close-approach configuration of idealized, weakly twisted right-handed filaments, displaying a left-handed arrangement (see movie S1) (*26*). (**C**) Same as (B) for the case of strongly twisted right-handed filaments, illustrating their entropic preference for right-handed arrangements (see movie S2) (*26*). (**D**) Same as (A) for the case of thermalized filaments. Markers denote experimental measurements [from (*21*)]. (**E**) Angular configuration minimizing the chiral two-body potential of mean force for thermalized 1x-lh origamis (see fig. S1 and section S2), illustrating the predominance of long-wavelength backbone fluctuations over local axial twist in their LChLC assembly.

In the absence of electrostatic interactions, the entropy-induced ordering of ground-state filaments is governed by their axial twist, which is found to stabilize antichiral LChLC phases, having opposite handedness with respect to the origami twist (Fig. 2A). This seemingly counterintuitive observation is explained by the fact that the pair excluded volume of weakly twisted, rod-like filaments is generally minimized by opposite-handed arrangements (Fig. 2B) (*24*). Conversely, this entropic preference is reversed in the case of strongly twisted filaments (Fig. 2C), which accounts for the weak right-handed phase predicted for the untwisted (s) origamis in terms of the intrinsic right-handed helicity of DNA (*27*). These findings mirror recent results on the LChLC assembly of continuously threaded particles, for which the quantitative validity of these simple geometric arguments has been investigated in detail (*26*).

However, these predictions are at odds with the experimental measurements of (*21*), which instead revealed a general tendency of origami filaments to stabilize isochiral LChLC phases, bearing the same handedness as their axial twist. Previous theoretical studies of DNA assemblies have attempted to attribute similar discrepancies to a potential antagonistic influence of electrostatic interactions (*27*, *29*, *30*), although the validity of this argument has been disputed by detailed numerical investigations (*31*). Here, we instead report that the main effect of the inclusion of the longer-ranged Debye-Hückel repulsion is to simply unwind the predicted cholesteric pitches by partially screening the chiral nucleotide distribution on the filament surface (see fig. S1 and section S2). This finding mirrors the conclusions of (*32*) for the LChLC behavior of bacterial cellulose microcrystals, whose twisted molecular morphologies closely resemble those of the origami ground states, and is consistent with recent all-atom simulations of short B-DNA oligomers, which failed to uncover a statistically significant chiral contribution attributable to electrostatics in DNA-DNA intermolecular interactions (*31*). Thus, these observations suggest that simple steric and electrostatic repulsion between ground-state filament conformations cannot account for either the handedness or the magnitude of their experimental cholesteric pitches.

### Role of conformational statistics

To assess the influence of conformational statistics on their cholesteric ordering, we make further use of the oxDNA model (*28*) to probe the detailed thermal fluctuations of the origami filaments. As in (*33*), we extend our theoretical framework to flexible particles through its combination with the numerical sampling of the filament conformational space by single-origami molecular dynamics (MD) simulations (see Materials and Methods). This hybrid approach, based on the Fynewever-Yethiraj density functional theory (*34*), has been shown to be quantitatively accurate in dilute assemblies of long and stiff persistent chains, for which the effects of many-particle interactions on conformational statistics are limited (see section S1) (*33*). This description is therefore well suited for our purposes, given the large persistence length (*l*_p_) of the origami structures [*l*_p_/*l*_c_ ≳ 5 (*35*)] and the low packing fractions of their stable blue LChLC phases (*21*). Notably, despite its experimental relevance, this regime of large but finite-particle rigidity (σ ≪ *l*_c_ ≲ *l*_p_) may not be easily probed by previous theories of cholesteric order, which either neglect the effects of flexibility altogether (*29*, *27*, *36*) or focus on semiflexible polymers in the coil limit (σ ≪ *l*_p_ ≪ *l*_c_), effectively treated as contiguous collections of rigid chiral segments (*37*).

Our results display an unexpected phase handedness inversion compared to the LChLC behavior of the origami ground states and a considerable tightening of the corresponding equilibrium pitches (Fig. 2D). The conjunction of these two factors allows for a convincing overall agreement with the experimental measurements of (*21*), albeit with a slight offset in the crossover value of the origami twist at which the phase handedness inversion occurs. These effects stem from the emergence of long-wavelength helical deformation modes along the backbone of thermalized origamis, which dominate the chiral component of their potential of mean force over the local surface chirality arising from axial twist (Fig. 2E and fig. S1).

**Fig. 3:**
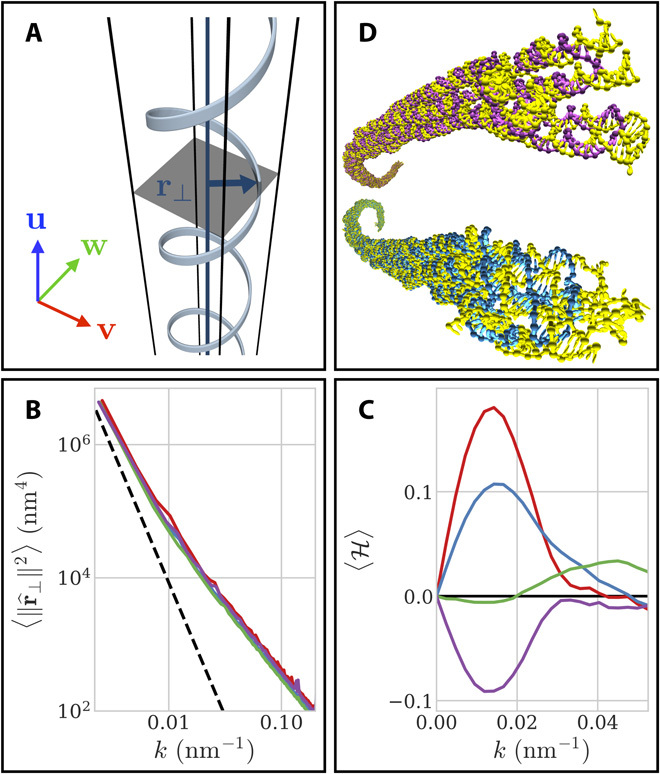
Conformational fluctuations and solenoidal writhe. (**A**) Transverse deformation vector **r**_⊥_ for an arbitrary backbone conformation. (**B**) Transverse fluctuation spectrum of each origami variant. The dashed line represents the theoretical scaling behavior of generic semiflexible filaments with bending rigidity *l*_p_/*l*_c_ = 8 at low deformation wave numbers (*k*) (see section S5). Colors are as in Fig. 2. (**C**) Net backbone helicity (〈H〉) as a function of *k*. Positive (resp. negative) values denote a statistical bias toward right-handed (resp. left-handed) deformation modes. The data are smoothed using a Savitzky-Golay filter of order 9 to facilitate visualization (*42*). Colors are as in Fig. 2. (**D**) Example simulated conformations of 1x-rh (top) and 1x-lh (bottom) origamis, respectively, displaying characteristic left- and right-handed backbone helicities.

This long-ranged, super-helical (or solenoidal) writhe may be quantified by Fourier analysis of the filament backbone conformations (Fig. 3A; see Materials and Methods). The transverse fluctuation spectra obtained using the oxDNA model are found to be consistent with the asymptotic scaling behavior of persistent chains in the limit of long-wavelength deformations for typical experimental values of the filament bending rigidity (Fig. 3B) (*21*). In this regime, the net backbone helicity of each origami variant is found to bear the opposite handedness to the axial twist of its ground state, with left-handed (right-handed) filaments predominantly favoring right-handed (left-handed) helical conformations, respectively (Fig. 3, C and D). Note that the antisymmetric character of the helicity measure H(*k*) (see Materials and Methods) imposes that H(0) = 0 and leads to the observation of a peak for H in Fig. 3C at the smallest accessible deformation wave number *k*_min_ = 1/*l*_c_. This peak should therefore be regarded as an effect of the finite size of the filaments and may a priori not be interpreted as evidence of a physical length scale identifying a preferred specific helical pitch for the origami shape fluctuations.

The geometric argument of Fig. 2B, applied to systems of weakly curled helices, predicts these conformations to display an entropic preference for opposite-handed arrangements (*26*). In this case, the stabilization of isochiral phases of twisted origami filaments therefore arises from their propensity for long-ranged, antichiral deformations under the effects of thermal fluctuations. This original chirality amplification mechanism is further evidenced by the relative insensitivity of our results to the inclusion of electrostatic interactions (Fig. 2D), as the typical length scales of the resulting backbone helicities are considerably larger than the experimental Debye screening length (λ_D_ ≃ 0.6 nm) (Fig. 3, C and D) (*21*).

### Ground-state structure and helical fluctuations

The origin of this fluctuation-stabilized solenoidal writhe, and of its dependence on filament twist, lies in the geometric constraints imposed by interhelical crossovers in the origami design. In the untwisted origami (s), the crossover separation is designed to exactly match the DNA pitch, so that crossovers between adjacent helices are separated by 21 bp—i.e., two full helical turns. In the left-handed designs, for instance, the number of base pairs between crossovers in certain sections of the filaments is reduced by one (known as a “deletion”). Assuming the origamis to be straight and untwisted, the resulting overtwist of the individual duplexes is given in the “initial” column of Table 1. The stress arising from this overtwist in the duplexes may be reduced if the origami as a whole adopts a left-twisted configuration about its long axis, as this reduces the net duplex twist between junctions (*22*). This redistribution of the stress leads to a decrease in the overtwist in the duplexes of roughly 30% upon going from the “initial” untwisted origami to the ground state (Fig. 1), as shown in Table 1.

The residual overtwist of the duplexes is further found to be substantially reduced by the thermal fluctuations. This is achieved by the origamis preferentially adopting writhed configurations with the same sign as the twist stress in the duplexes (see Table 1 and section S6). In other words, when left-handed origamis fluctuate to bear a right-handed helical writhe, the elastic cost of bending is partially offset by a reduction in the residual overtwist of the DNA helices, while left-handed backbone conformations are energetically penalized by a further overwinding of the duplexes. Conversely, in the case of right-twisted origamis, the required base pair insertions lead to an underwinding of the individual DNA helices, which, in turn, favors a left-handed solenoidal writhe.

The observed offset in the filament phase handedness inversion behavior, apparent in Fig. 2D, could thus be partially explained in terms of a small misestimate of the equilibrium duplex twist density Tw_0_, as the equilibrium helical pitch of B-DNA within constrained origami structures may slightly differ from the unconfined value 1/Tw_0_ ≃ 10.5 bp assumed in both the computation of the origami ground states (*23*) and the parameterization of the oxDNA model (*28*). Additional possible sources of error include other potential shortcomings of the oxDNA model, such as our use of sequence-averaged mechanics for DNA, or the limitation of soft nonbonded interactions to simple Debye-Hückel electrostatics (*28*). The overestimations in the magnitude of our cholesteric pitch predictions (Fig. 2D) are further consistent with the symmetry limitations of the theory, in which long-ranged biaxial correlations arising from broken local cylindrical invariance are neglected (*25*). The limited extent of these discrepancies, relative to the vast gap between molecular and cholesteric length scales, combined with the satisfactory experimental agreement achieved in terms of isotropic/cholesteric binodal concentrations (Table 2) and in the magnitude of the underlying macroscopic curvature elasticities (see fig. S2 and section S3), nonetheless evidences the ability of the theory to correctly capture the basic physics of LChLC assembly in our case.

**Table 1:** Duplex twist and backbone writhe in thermalized and unthermalized origamis. The overtwist density ΔTw ≡ Tw − Tw_0_ is averaged over the six constituent duplexes of each origami design (see Materials and Methods). The “initial” column corresponds to values obtained by assuming that the origamis adopt a straight and untwisted conformation. The writhe density Wr of the origami centerlines is smaller than the last significant digit for all “initial” and ground-state filaments and is therefore only reported in the case of the thermalized systems. All thermalized values are further averaged over the full ensemble of simulated conformations for each origami variant.

**State**	**Initial**	**Ground**	**Thermalized**	
**Design**	**ΔTw (turns/μm)**	**ΔTw (turns/μm)**	**〈ΔTw〉 (turns/μm)**	**〈Wr〉 (mm^−1^)**
2x-lh	15.07	10.08	4.05	73.3
1x-lh	7.00	4.57	3.20	37.6
s	0	0	0.61	−6.1
1x-rh	−6.42	−4.16	−2.72	−38.4

**Table 2:** Isotropic/cholesteric coexistence concentrations for thermalized untwisted origamis. *c*_st + el_ and *c*_st_ denote the theoretical predictions obtained by taking into account steric interparticle repulsion with and without electrostatic interactions, respectively (see Materials and Methods). Results are compared with the experimental measurements of (*21*).

**Binodal**	***c*_st_**	***c*_st+el_**	**(*21*)**
Isotropic	31.8 g/liter	28.3 g/liter	28 g/liter
Cholesteric	36.7 g/liter	32.2 g/liter	37 g/liter

## DISCUSSION

We have presented the successful application of an extended Onsager theory to the quantitative description of LChLC order in systems of long DNA origami filaments. Its combination with an accurate conformational sampling scheme demonstrates that phase chirality in this case results from the weak, fluctuation-stabilized solenoidal writhing of the filament backbones and is therefore largely governed by intramolecular mechanics. These long-wavelength, chiral deformation modes, which dominate cholesteric assembly over the much shorter length scales associated with the twisted morphology of the ground state, are further shown to be linked to the ground-state structure in a nontrivial fashion, as illustrated by the stabilization of antichiral deformation modes through twist-writhe conversion of the filament elastic energy.

The net helicity of these backbone fluctuations is found to originate from the weak over- or underwinding of the constituent duplexes in the origami ground states. Similar geometrical frustration phenomena have been shown to widely regulate equilibrium morphology in cohesive bundles of generic chiral filaments (*38*), which may be found in the molecular structure of a number of flexible cholesteric mesogens, ranging from amyloid fibrils (*39*) to the protein coat of filamentous viruses (*11*). The LChLC assembly of these colloids could therefore be expected to be similarly affected by potential solenoidal deformation modes, thus giving credence to the hypothetical “corkscrew model” first proposed in (*11*) to explain the puzzling cholesteric behavior of virus suspensions. This chirality amplification mechanism represents a marked shift from the prevailing theoretical models, in which the macroscopic breaking of mirror symmetry has generally been attributed to the intermolecular interactions arising from the chiral morphology of the molecular ground state (*14*, *27*, *36*), and more broadly suggests a novel self-assembly paradigm for LChLCs in which subtle, long-ranged conformational features—rather than local chemical structure—dictate macroscopic chiral organization.

Last, the current study, together with the experiments of (*21*), may provide a new framework to systematically explore the link between molecular properties and supramolecular organization and illustrates how the unique ability of DNA origamis to assemble into programmable shapes of near-arbitrary complexity may be fruitfully combined with the capacity of our theoretical description to rationalize their phase behavior, to elucidate the hierarchical self-assembly of complex, chiral macroscopic materials.

## MATERIALS AND METHODS

### MD simulation setup

Single-origami simulations were run for each of the six-helix bundle designs in (*21*) using the oxDNA coarse-grained model, which represents DNA as a collection of rigid nucleotides interacting through excluded volume, Debye-Hückel, stacking, and hydrogen- and covalent-bonding potentials (*28*). Calculations were performed on graphics processing units (GPUs) in the canonical ensemble using an Andersen-like thermostat and sequence-averaged DNA thermodynamics, assuming room temperature conditions (*T* = 293 K) and fixed monovalent salt concentration *c*_Na^+^_ = 0.5 M. This value was chosen in slight excess of the experimental salt concentration *c*_Na^+^_ = 0.26 M (*21*), used throughout the rest of the paper, to limit computational costs. The effects of this approximation on origami conformational statistics are expected to be minimal in the context of the simplified oxDNA treatment of electrostatics (*28*). Relaxation was achieved through equilibration runs of O(10^6^) MD steps starting from the origami ground state, and production runs of O(10^9^) steps, were conducted to generate O(10^3^) uncorrelated conformations for each origami variant. The statistical independence of the resulting conformations was assessed by ensuring the vanishing autocorrelation of their end-to-end separation distance.

### Conformational analysis

The discretized origami backbones are obtained by averaging the center-of-mass locations of their bonded nucleotides over the six constituent duplexes within each transverse plane along the origami contour (*22*). We define the molecular frame R = [**u v w**] of each conformation as the principal frame of its backbone gyration tensor, such that **u** and **v** correspond to the respective direction of maximum and minimum dispersion of the origami backbone (*33*). Shape fluctuations are described by the contour variations of the transverse position vector

$$r_{⊥}(s)=r(s)-r_{u}(s)u$$ (1)

with **r**(*s*) the position of the discretized backbone segment with curvilinear abscissa *s* and *r_u_*(*s*) ≡ **r**(*s*) · **u**, assuming the backbone center of mass to be set to the origin of the frame. Denoting by Δ*s* the curvilinear length of each segment, the Fourier components of **r**_⊥_ read as

$${\hat{r}}_{⊥}(k)=\sum_{s}\Delta s\;r_{⊥}(s)\times e^{-2\text{iπks}}$$ (2)

Using the convolution theorem, the spectral coherence between the two transverse components of an arbitrary backbone deformation mode may be quantified by their Fourier-transformed cross-correlation function ${\hat{c}}_{\mathrm{vw}}$

$${\hat{c}}_{\mathrm{vw}}(k)={\hat{r}}_{⊥v}(k)\times {\hat{r}}_{⊥w}^{*}(k)$$ (3)

where ${\hat{r}}_{⊥x}={\hat{r}}_{⊥}\cdot x$ for **x** ∈ {**v**, **w**} and ${\hat{r}}_{⊥w}^{*}$ is the complex conjugate of ${\hat{r}}_{⊥w}$. It is shown in section S4 that a helicity order parameter H(*k*) for a deformation mode with arbitrary wave number *k* about the filament long axis **u** may be derived in the form

$$H(k)=\frac{2\times I\{{\hat{c}}_{\mathrm{vw}}(k)\}}{{\hat{c}}_{\mathrm{vv}}(k)+{\hat{c}}_{\mathrm{ww}}(k)}$$ (4)

with $I\{{\hat{c}}_{\mathrm{vw}}\}$ the imaginary part of ${\hat{c}}_{\mathrm{vw}}$. One may check that −1 ≤ H(*k*) ≤ 1, with H(*k*) = ± 1 if and only if the two transverse Fourier components bear equal amplitudes and lie in perfect phase quadrature. In this case, ${\hat{r}}_{⊥}(k)$ describes an ideal circular helical deformation mode with pitch 1/*k* and handedness determined by the sign of H.

### Determination of duplex twist and backbone writhe

Let **r**_*i*,1_(*s_i_*) and **r**_*i*,2_(*s_i_*) be a set of continuous curves interpolating the positions of the nucleotide centers of mass of the *i*th constituent duplex of an arbitrary origami conformation. The unit tangent and normal vectors **t***_i_* and **n***_i_* at a given curvilinear abscissa *s_i_* respectively read as

$$\begin{array}{c} t_{i}(s_{i})\equiv \frac{dr_{i}(s_{i})}{ds_{i}}\\ n_{i}(s_{i})\equiv \frac{r_{i,1}(s_{i})-r_{i,2}(s_{i})}{∥r_{i,1}(s_{i})-r_{i,2}(s_{i})∥} \end{array}$$

where **r***_i_* ≡ (**r**_*i*,1_ + **r**_*i*,2_)/2 is the continuous duplex centerline with contour length *l_i_*. The average twist density Tw of each duplex may then be obtained from the sum of the local stacking angles between consecutive base pairs (*40*)

$${\text{Tw}}_{i}=\frac{1}{2\pi l_{i}}\int_{0}^{l_{i}}{\mathrm{ds}}_{i}\;t_{i}(s_{i})\cdot \{n_{i}(s_{i})\times \frac{dn_{i}(s_{i})}{{\mathrm{ds}}_{i}}\}$$ (5)

For stiff origamis, whose centerline curve $r\equiv \sum_{i=1}^{6}r_{i}/6$ does not display any turning points in **u** (*dr_u_*/*ds* > 0), the so-called polar writhe Wr of the filament backbone simply reduces to the local contribution (*41*)

$$\text{Wr}=\frac{1}{2\pi l_{c}}\int_{0}^{l_{c}}\mathrm{ds}\frac{u\cdot \{t(s)\times \frac{dt(s)}{\mathrm{ds}}\}}{1+u\cdot t(s)}$$ (6)

where **t** ≡ *d***r**/*ds* is the unit backbone tangent vector. It may then be shown that Wr > 0 (resp. Wr < 0) if **t** winds about **u** in a right-handed (resp. left-handed) fashion (*41*). Equations 5 and 6 are evaluated numerically through standard quadrature methods, using cubic spline interpolations for all discrete curves (*40*). A Savitzky-Golay filter of order 9 (*42*) was preliminarily applied to the backbone curve to weed out irrelevant short-wavelength contour fluctuations arising from our geometric definition of the origami centerline (*40*). The last 𝒪(10) base pair planes at each of the filament extremities were excluded from the calculations to limit the influence of end effects.

### Molecular theory of cholesteric order

We consider a cholesteric phase of director field **n** and helical axis **e***_z_* in the laboratory frame R_lab_ ≡ [**e***_x_* **e***_y_* **e***_z_*], whose continuum Helmholtz free energy density is expressed by the Oseen-Frank functional (*43*)

$$f=f_{0}+\frac{1}{2}\{K_{2}{\left(n\cdot [\nabla \times n]\right)}^{2}+2k_{t}(n\cdot [\nabla \times n])\}$$ (7)

Given the high anisotropy of the origami structures and the low packing fractions marking the onset of their LChLC organization (*21*), the mean-field free energy *f*_0_ of their reference nematic state with uniform director **n** ≡ **e***_x_* may be written in a generalized Onsager form, based on the second-virial kernel κ (*34*) (see section S1)

$$\kappa (\theta ,\theta \prime )=\int dr_{12}∯dR_{1}dR_{2}\;\bar{f}(r_{12},R_{1},R_{2})\delta (\text{cos}\;\theta_{1}-\text{cos}\;\theta )\delta (\text{cos}\;\theta_{2}-\text{cos}\;\theta \prime )$$ (8)

with δ the Dirac distribution and $\bar{f}$ the Mayer *f*-function averaged over all pairs of accessible molecular conformations

$$\bar{f}(r_{12},R_{1},R_{2})=\langle \langle e^{-\beta U_{\text{inter}}(r_{12},R_{1},R_{2})}-1\rangle \rangle$$ (9)

In Eq. 9, *U*_inter_(**r**_12_, R_1_, R_2_) denotes the intermolecular interaction energy of two arbitrary origami conformations with center-of-mass separation **r**_12_ and respective molecular-frame orientations R_1,2_, and 〈·〉 is the ensemble average over the single-origami conformations generated by MD simulations (*33*). Local uniaxial order is described by the equilibrium orientation distribution function ψ(cos θ) ≡ ψ(**e***_x_* · **u**), quantifying the dispersion of the origami long axes **u** = R · **e***_x_* about **e***_x_*. ψ is obtained by functional minimization of *f*_0_ at fixed number density ρ and inverse temperature β = 1/*k*_B_*T* (*25*)

$$\psi (\text{cos}\;\theta )=\frac{1}{Z}\text{exp}\;\{\frac{\rho }{4\pi^{2}}\int_{-1}^{1}d\text{cos}\;\theta^{\prime }\;\psi (\text{cos}\;\theta^{\prime })\kappa (\theta ,\theta^{\prime })\}$$ (10)

with *Z* a Lagrange multiplier ensuring the normalization of ψ. The Oseen-Frank twist elastic modulus *K*_2_ and chiral strength *k_t_* read as (see section S1)

$$\beta K_{2}=\frac{\rho^{2}}{2}\int_{V}dr_{12}∯dR_{1}dR_{2}\;\bar{f}(r_{12},R_{1},R_{2})\times \dot{\psi }(\text{cos}\;\theta_{1})\dot{\psi }(\text{cos}\;\theta_{2})r_{z}^{2}u_{1y}u_{2y}$$ (11)

$$\beta k_{t}=\frac{\rho^{2}}{2}\int_{V}dr_{12}∯dR_{1}dR_{2}\;\bar{f}(r_{12},R_{1},R_{2})\times \psi (\text{cos}\;\theta_{1})\dot{\psi }(\text{cos}\;\theta_{2})r_{z}u_{2y}$$ (12)

with *r_z_* = **r**_12_ · **e***_z_*, *u_iy_* = **u***_i_* · **e***_y_* and $\dot{\psi }$ the first derivative of ψ. The equilibrium cholesteric pitch is determined by the competition between chiral torque and curvature elasticity and is obtained by minimization of the elastic contribution to the free energy density *f* (Eq. 7) (*24*)

$$P=2\pi \frac{K_{2}}{k_{t}}$$ (13)

Equations 8, 11, and 12 are evaluated through optimized virial integration techniques (*26*) over 16 independent runs of 10^13^ Monte Carlo (MC) steps, using oxDNA-parameterized Debye-Hückel and steric internucleotide repulsion for the intermolecular potential *U*_inter_ (*28*). The conformational average in Eq. 9 is performed by stochastic sampling over the simulated origami conformations in Eqs. 8, 11, and 12 (*33*). Equation 10 is solved through standard numerical means (*44*). Convergence was ensured by verifying the numerical dispersion of the computed pitches (Eq. 13) to be less than 10% across the results of the 16 MC runs, using independent bootstrap samples of the ensemble of simulated conformations. Binodal points were calculated by equating chemical potentials and osmotic pressures in the isotropic and cholesteric phase and numerically solving the resulting coupled coexistence equations (*25*). Mass concentrations were obtained, assuming a molar weight of 650 Da per base pair.

## Supplementary Material

aaw8331_Movie_S2.mp4

aaw8331_Movie_S1.mp4

aaw8331_SM.pdf

## SUPPLEMENTARY MATERIALS

Supplementary material for this article is available at http://advances.sciencemag.org/cgi/content/full/6/31/eaaw8331/DC1
