# Supplementary material for: Chiral shape fluctuations and the origin of chirality in cholesteric phases of DNA origamis
Source: Sci Adv. 2020 Jul 29;6(31):eaaw8331. doi: 10.1126/sciadv.aaw8331 (PMC7399560; doi:10.1126/sciadv.aaw8331)
Supplement: aaw8331_SM.pdf [file aaw8331_SM.pdf]

Science Advances
AAAS

advances.sciencemag.org/cgi/content/full/6/31/eaaw8331/DC1

Supplementary Materials for

Chiral shape fluctuations and the origin of chirality in cholesteric phases of DNA origamis

Maxime M. C. Tortora*, Garima Mishra, Domen Prešern, Jonathan P. K. Doye

*Corresponding author. Email: maxime.tortora@ens-lyon.fr

Published 29 July 2020, *Sci. Adv.* **6**, eaaw8331 (2020)
DOI: 10.1126/sciadv.aaw8331

The PDF file includes:

Sections S1 to S6
Figs. S1 and S2
Legends for movies S1 and S2
References

Other Supplementary Material for this manuscript includes the following:

(available at advances.sciencemag.org/cgi/content/full/6/31/eaaw8331/DC1)

Movies S1 and S2

S1 Fynewever-Yethiraj density functional theory for LChLCs

In the context of classical density functional theory, the Helmholtz free energy of a system of polyatomic molecules may be written in the general form (*45*)

$$\mathscr{F}[\rho_m] = \mathscr{F}_{\text{id}}[\rho_m] + \mathscr{F}_{\text{ex}}[\rho_m], \tag{1}$$

where the microscopic density ρ_m generally depends on the discrete set of atom positions $\{\mathbf{r}_i\}_{i\geq 1}$ and bond orientations $\{\mathcal{R}_j\}_{j\geq 1}$ characterising the full microscopic state of each individual constituent particle. The center-of-mass position $\mathbf{r}$ and molecular orientation $\mathcal{R}$ of a given particle in any conformation are uniquely determined by the specification of all internal degrees of freedom $\{\mathbf{r}_i\}$ and $\{\mathcal{R}_j\}$, so that one may write, without loss of generality,

$$\rho_m\big(\{\mathbf{r}_i\}, \{\mathcal{R}_j\}\big) = \rho_m\big(\mathbf{r}, \mathcal{R}, \{\mathcal{X}\}\big), \tag{2}$$

with $\{\mathcal{X}\} \equiv \big(\{\mathbf{r}_i\}_{i\geq 2}, \{\mathcal{R}_j\}_{j\geq 2}\big)$. Let $\mathbf{r}'_i$ and $\mathcal{R}'_j$ be the respective projections of $\mathbf{r}_i$ and $\mathcal{R}_j$ in the molecular frame $\mathcal{R}$ centerd on $\mathbf{r}$,

$$\mathbf{r}'_i \equiv \mathcal{R}^\mathsf{T} \cdot (\mathbf{r}_i - \mathbf{r}), \tag{3}$$

$$\mathcal{R}'_j \equiv \mathcal{R}^\mathsf{T} \cdot \mathcal{R}_j, \tag{4}$$

with $\mathcal{R}^\mathsf{T}$ the matrix transpose of $\mathcal{R}$. The Fynewever-Yethiraj (FY) approximation postulates that ρ_m may be cast in the decoupled form (*33, 34, 46*)

$$\rho_m\big(\mathbf{r}, \mathcal{R}, \{\mathcal{X}\}\big) \simeq \rho(\mathbf{r}, \mathcal{R}) \times P\big(\{\mathcal{X}'\}\big), \tag{5}$$

where $\{\mathcal{X}'\} \equiv \big(\{\mathbf{r}'_i\}_{i\geq 2}, \{\mathcal{R}'_j\}_{j\geq 2}\big)$. In Eq. (5), ρ corresponds to the *molecular density* describing the global distribution of particle centers of mass $\mathbf{r}$ and orientations $\mathcal{R}$ throughout the sample, while P quantifies the distribution of the conformational degrees of freedom $\mathbf{r}'_i$ and $\mathcal{R}'_j$ in the local molecular frame, subject to the respective normalisation constraints (*46*)

$$\int d\{\mathcal{X}'\}\, P\big(\{\mathcal{X}'\}\big) = 1, \tag{6}$$

$$\int_V d\mathbf{r} \oint d\mathcal{R}\, \rho(\mathbf{r}, \mathcal{R}) = N. \tag{7}$$

In the FY theory, P is assumed to be entirely determined by the intra-molecular interaction potential $U_{\text{intra}} = U_{\text{intra}}\big(\{\mathcal{X}'\}\big)$, so as to be independent of the overall position $\mathbf{r}$ and orientation $\mathcal{R}$ of the molecule. In the absence of external fields, this approximation amounts to neglecting the effects of many-particle interactions on conformational statistics, and is therefore only rigorously justifiable in the case of highly-stiff molecules, for which the accessible conformational space is largely independent of density in the regime of low-to-moderate particle packing fractions (*33*).

Discarding the effects of inter-molecular interactions, the *ideal* component $\mathscr{F}_{\text{id}}$ of the Helmholtz

free energy functional is given by (*46, 47*)

$$\beta\mathscr{F}_{\mathrm{id}}[\rho_m] = \int_V d\mathbf{r} \oint d\mathcal{R} \int d\{\mathcal{X}\}\, \rho_m\big(\mathbf{r}, \mathcal{R}, \{\mathcal{X}\}\big) \\ \times \Big\{ \log\big[\lambda_{\mathrm{dB}}^3 \rho_m\big(\mathbf{r}, \mathcal{R}, \{\mathcal{X}\}\big)\big] - 1 + \beta U_{\mathrm{intra}}\big(\{\mathcal{X}\}\big) \Big\},$$

with λ_{dB} the thermal de Broglie wavelength. Using Eqs. (5)–(7),

$$\beta\mathscr{F}_{\mathrm{id}}[\rho] = \int_V d\mathbf{r} \oint d\mathcal{R}\, \rho(\mathbf{r}, \mathcal{R}) \big\{ \log\big[\lambda^3 \rho(\mathbf{r}, \mathcal{R})\big] - 1 \big\}, \tag{8}$$

where the lengthscale λ now reads as

$$\lambda = \lambda_{\mathrm{dB}} \exp\left\{ \frac{1}{3} \int d\{\mathcal{X}'\}\, P\big(\{\mathcal{X}'\}\big) \Big[\log P\big(\{\mathcal{X}'\}\big) + \beta U_{\mathrm{intra}}\big(\{\mathcal{X}'\}\big) \Big] \right\},$$

in which we used the change of variables of Eqs. (3) and (4), with unit Jacobian determinant. Note that λ generally depends on intra-molecular properties as well as temperature, but is independent of ρ. In the case of a prolate nematic phase with arbitrary director field $\mathbf{n}(\mathbf{r})$, the molecular density function ρ takes the form

$$\rho(\mathbf{r}, \mathcal{R}) = \overline{\rho}\psi\big\{\mathbf{u} \cdot \mathbf{n}(\mathbf{r})\big\}, \tag{9}$$

where $\overline{\rho} \equiv N/V$ is the molecular number density, and the orientation distribution function (ODF) ψ describes the ordering of the long molecular axes $\mathbf{u} \equiv \mathcal{R} \cdot \mathbf{e}_x$ about the local director $\mathbf{n}(\mathbf{r})$. Note that Eq. (9) is only valid in the limit where the spatial fluctuations of $\mathbf{n}$ are negligible at the molecular lengthscale, as is typical in experimental cholesterics, and in the absence of long-ranged biaxial correlations, as is commonly presumed in theoretical studies (*14–16, 25, 48*). Eq. (9) further assumes the local molecular density $\overline{\rho}$ to be unaffected by director fluctuations, which is expected to be appropriate in the case of the twist deformations characteristic of LChLCs (*49*). Let us define the unit-Jacobian transformation

$$\mathcal{R}' \equiv \mathcal{T}(\mathbf{r})^{\mathsf{T}} \cdot \mathcal{R}, \tag{10}$$

with $\mathcal{T}(\mathbf{r})$ a rotation matrix such that

$$\mathbf{n}(\mathbf{r}) = \mathcal{T}(\mathbf{r}) \cdot \mathbf{n}(\mathbf{0}) \equiv \mathcal{T}(\mathbf{r}) \cdot \mathbf{n}_0. \tag{11}$$

Eqs. (8) and (9) immediately yield

$$\frac{\beta \mathscr{F}_{\text{id}}[\psi]}{V} = 4\pi^2 \rho \int_{-1}^{1} du'_x \, \psi(u'_x) \Big\{ \log \left[\rho \lambda^3 \psi(u'_x) \right] - 1 \Big\}, \tag{12}$$

where $u'_x \equiv \mathbf{n}_0^\top \cdot \mathcal{R}' \cdot \mathbf{e}_x$, and we dropped the overline notation from $\overline{\rho}$. Thus, Eq. (12) indicates that $\mathscr{F}_{\text{id}}$ is independent of the configuration of the director field $\mathbf{n}$.

In the case of highly stiff and elongated molecules, the *excess* component $\mathscr{F}_{\text{ex}}$ of the Helmholtz free energy may be related to the inter-molecular interaction potential U_{inter} at the second virial level through the Onsager mean-field functional (*47*),

$$\beta \mathscr{F}_{\text{ex}}[\rho_m] = -\frac{1}{2} \iint d\mathbf{1} d\mathbf{2} \, \rho_m(\mathbf{1}) \rho_m(\mathbf{2}) f(\mathbf{1}, \mathbf{2}), \tag{13}$$

where the shorthand $\mathbf{i} \equiv \big(\mathbf{r}_i, \mathcal{R}_i, \{\mathcal{X}_i\} \big)$ refers to the full microscopic degrees of freedom associated with particle i, and f is the so-called Mayer function,

$$f(\mathbf{1}, \mathbf{2}) \equiv \exp \big\{ -\beta U_{\text{inter}}(\mathbf{1}, \mathbf{2}) \big\} - 1.$$

Using Eqs. (5) and (9), Eq. (13) may be recast as (*25, 33, 46*)

$$\beta \mathscr{F}_{\text{ex}}[\psi] = -\frac{\rho^2}{2} \iint_V d\mathbf{r}_1 d\mathbf{r}_2 \oiint d\mathcal{R}_1 d\mathcal{R}_2 \, \psi\{\mathbf{u}_1 \cdot \mathbf{n}(\mathbf{r}_1)\} \psi\{\mathbf{u}_2 \cdot \mathbf{n}(\mathbf{r}_2)\} \overline{f}(\mathbf{r}_1, \mathbf{r}_2, \mathcal{R}_1, \mathcal{R}_2), \tag{14}$$

with $\overline{f}$ the *conformational average* of the Mayer function,

$$\overline{f}(\mathbf{r}_1, \mathbf{r}_2, \mathcal{R}_1, \mathcal{R}_2) \equiv \iint d\{\mathcal{X}'_1\} d\{\mathcal{X}'_2\} \, P\big(\{\mathcal{X}'_1\}\big) P\big(\{\mathcal{X}'_2\}\big) f(\mathbf{1}, \mathbf{2}). \tag{15}$$

Note that the integrand in Eq. (14) is non-zero only if $\overline{f}(\mathbf{r}_1, \mathbf{r}_2, \mathcal{R}_1, \mathcal{R}_2) \neq 0$, i.e., if there exists two molecular conformations with respective center-of-mass positions $\mathbf{r}_{1,2}$ and overall orientations $\mathcal{R}_{1,2}$ such that $U_{\text{inter}} \neq 0$. It follows that in the case of short-range interaction

potentials, a pair of molecules 1 and 2 may physically contribute to the integral in Eq. (14) only if their center-of-mass separation distance $\mathbf{r}_{12} \equiv \mathbf{r}_2 - \mathbf{r}_1$ is of the order of the typical molecular dimensions. Let us introduce the particle barycenter $\mathbf{R} = (\mathbf{r}_1 + \mathbf{r}_2)/2$,

$$\mathbf{r}_{1,2} = \mathbf{R} \mp \frac{\mathbf{r}_{12}}{2}. \tag{16}$$

Under the assumptions of Eq. (9), we may thus write (*15*)

$$\mathbf{n}(\mathbf{r}_i) \simeq \mathbf{n}(\mathbf{R}) \mp \frac{\nabla \mathbf{n}(\mathbf{R}) \cdot \mathbf{r}_{12}}{2} + \frac{\nabla^2 \mathbf{n}(\mathbf{R}) : (\mathbf{r}_{12} \otimes \mathbf{r}_{12})}{8}.$$

with $\otimes$ and $:$ the respective tensor and double dot products. In the case of a cholesteric phase of axis $\mathbf{e}_z$ and inverse pitch $q \equiv 2\pi/\mathcal{P}$, the helical modulation of the director field takes the form

$$\mathbf{n}(\mathbf{R}) = \cos(qR_z)\,\mathbf{e}_x + \sin(qR_z)\,\mathbf{e}_y,$$

where $R_z \equiv \mathbf{R} \cdot \mathbf{e}_z$ and we have chosen the laboratory frame such that $\mathbf{e}_x \equiv \mathbf{n}_0$. Let $\mathcal{T}(\mathbf{R}) \equiv \left[\mathbf{e}'_x\ \mathbf{e}'_y\ \mathbf{e}'_z\right]$ be a local rotating frame satisfying Eq. (11),

$$\mathcal{T}(\mathbf{R}) \equiv \begin{bmatrix} \cos(qR_z) & -\sin(qR_z) & 0 \\ \sin(qR_z) & \cos(qR_z) & 0 \\ 0 & 0 & 1 \end{bmatrix}.$$

It is straightforward to show that

$$\nabla \mathbf{n}(\mathbf{R}) = q\,\mathbf{e}'_y \otimes \mathbf{e}'_z,$$

$$\nabla^2 \mathbf{n}(\mathbf{R}) = -q^2\,\mathbf{e}'_x \otimes \mathbf{e}'_z \otimes \mathbf{e}'_z,$$

which directly lead to

$$\psi\{\mathbf{u}_i \cdot \mathbf{n}(\mathbf{r}_i)\} = \psi(u'_{ix}) \mp \frac{q u'_{iy} r'_z}{2} \dot{\psi}(u'_{ix}) - \frac{u'_{ix}}{2}\left\{\frac{q r'_z}{2}\right\}^2 \dot{\psi}(u'_{ix}) + \frac{1}{2}\left\{\frac{q u'_{iy} r'_z}{2}\right\}^2 \ddot{\psi}(u'_{ix}) + \mathcal{O}(q^3), \tag{17}$$

where primed quantities are expressed in the rotating frame $\mathcal{T}(\mathbf{R})$, with $u'_{ij} \equiv \mathbf{u}_i \cdot \mathbf{e}'_j$ and $r'_z \equiv \mathbf{r}_{12} \cdot \mathbf{e}'_z$. Plugging Eq. (17) into Eq. (14), and using the changes of variables of Eqs. (10)

and (16), we obtain

$$\mathscr{F} = \int_V d\mathbf{R}\, f(\mathbf{R}) \equiv \int_V d\mathbf{R}\,(f_0 + f_d),$$

in which f_0 is the free energy density of the uniform nematic state with director $\mathbf{n}_0 \equiv \mathbf{e}_x$,

$$\beta f_0[\psi] = 4\pi^2\rho \int_{-1}^{1} du_x\, \psi(u_x)\Big\{ \log\left[\rho\lambda^3\psi(u_x)\right] - 1\Big\}$$
$$- \frac{\rho^2}{2}\int_V d\mathbf{r}_{12} \oiint d\mathcal{R}_1 d\mathcal{R}_2\, \psi(u_{1x})\psi(u_{2x})\overline{f}(\mathbf{r}_{12}, \mathcal{R}_1, \mathcal{R}_2), \quad (18)$$

where we used Eqs. (1) and (12), and dropped the prime notation from the dummy integration variables. The integration by parts of the second-order terms in Eq. (17) with respect to $\mathbf{R}$ yields the distortion free energy density f_d in the form, to quadratic order in q (*24*),

$$f_d[\psi] = -k_t[\psi]q + K_2[\psi]\frac{q^2}{2}, \quad (19)$$

which by term-to-term comparison with the Oseen-Frank free energy (Eq. (7) in the main text) leads to the microscopic expressions of the chiral strength k_t and twist elastic modulus K_2 as direct generalisations of the Poniewierski-Stecki formulae (*50*),

$$\beta K_2[\psi] = \frac{\rho^2}{2}\int_V d\mathbf{r}_{12} \oiint d\mathcal{R}_1 d\mathcal{R}_2\, \overline{f}(\mathbf{r}_{12}, \mathcal{R}_1, \mathcal{R}_2) \times \dot{\psi}(u_{1x})\dot{\psi}(u_{2x})u_{1y}u_{2y}r_z^2, \quad (20)$$

$$\beta k_t[\psi] = \frac{\rho^2}{2}\int_V d\mathbf{r}_{12} \oiint d\mathcal{R}_1 d\mathcal{R}_2\, \overline{f}(\mathbf{r}_{12}, \mathcal{R}_1, \mathcal{R}_2) \times \psi(u_{1x})\dot{\psi}(u_{2x})u_{2y}r_z, \quad (21)$$

from which one recovers Eqs. (9) and (10) of the main text, with $\cos\theta_i \equiv u_{ix}$.

In the limit of long-wavelength director distortions, it may be assumed that the degree of local orientational order is unaffected by the spatial variations of $\mathbf{n}$, so that the equilibrium ODF ψ_{eq} of the cholesteric phase may be assimilated to that ψ_{eq}^0 of the uniform nematic state. This approximation has been previously shown to be valid for cholesteric pitches as short as a few dozen particle diameters (*25*), and is expected to hold without restrictions in our case. ψ_{eq} may then be obtained through the functional minimisation of f_0 (Eq. (18)) in the self-consistent form

$$\psi_{\text{eq}}(u_x) = \frac{1}{Z}\exp\left\{\frac{\rho}{4\pi^2}\int_{-1}^{1} du'_x\, \psi_{\text{eq}}(u'_x)\kappa(u_x, u'_x)\right\},$$

with Z a Lagrange multiplier such that

$$4\pi^2 \int_{-1}^{1} du_x \, \psi_{\rm eq}(u_x) = 1,$$

and κ a generalised excluded-volume kernel (*34*),

$$\kappa(u_x, u'_x) = \int d\mathbf{r}_{12} \oiint d\mathcal{R}_1 d\mathcal{R}_2 \, \overline{f}(\mathbf{r}_{12}, \mathcal{R}_1, \mathcal{R}_2)\delta(u_{1x} - u_x)\delta(u_{2x} - u'_x). \tag{22}$$

As in Refs. (*33,34,46*), we sample the conformational distribution P by single-molecule simulations, following the numerical protocol described in the main text (see Materials and Methods). In this context, the Mayer function $\overline{f}$ (Eq. (15)) is averaged over all pairs of simulated origami conformations in the computation of Eqs. (20), (21) and (22), and the inverse equilibrium cholesteric pitch $q_{\rm eq}$ is finally obtained by minimisation of f_d at fixed T and ρ (Eq. (19)) (*24*),

$$q_{\rm eq}(\rho, T) = \frac{k_t[\psi_{\rm eq}]}{K_2[\psi_{\rm eq}]}.$$

S2 Chiral potential of mean force and phase handedness

In the following, let us denote the properties relative to right- and left-handed pair configurations by $+$ and $-$ subscripts, respectively. The angular two-body potential of mean force (PMF) $\overline{U}_\pm$ associated with two-particle arrangements of fixed handedness is given by (*15, 51*)

$$\beta\overline{U}_\pm(\theta) \equiv -\log \left\langle e^{-\beta U_{\rm inter}} \right\rangle_\pm^{(\theta)}, \tag{23}$$

where the configurational average $\left\langle \, \cdot \, \right\rangle_\pm^{(\theta)}$ is defined as

$$\left\langle e^{-\beta U_{\rm inter}} \right\rangle_\pm^{(\theta)} \equiv \frac{1}{V_{\rm int}} \int_V d\mathbf{r}_{12} \oiint d\mathcal{R}_1 d\mathcal{R}_2 \, e^{-\beta U_{\rm inter}(\mathbf{r}_{12}, \mathcal{R}_1, \mathcal{R}_2)} \times \delta(\mathbf{u}_1 \cdot \mathbf{u}_2 - \cos\theta)\Theta\big\{ \pm \mathbf{r}_{12} \cdot (\mathbf{u}_1 \times \mathbf{u}_2)\big\}, \tag{24}$$

using the notations of Section S1. In Eq. (24), the Heaviside function Θ mirrors the fact that the handedness of an arrangement of two particles with center-of-mass separation vector $\mathbf{r}_{12} \equiv$

$\mathbf{r}_2 - \mathbf{r}_1$ and respective long axes $\mathbf{u}_i \equiv \mathcal{R}_i \cdot \mathbf{e}_x$ is determined by the sign of $\mathbf{r}_{12} \cdot (\mathbf{u}_1 \times \mathbf{u}_2)$, and V_{int} represents the total volume spanned by the spatial and angular integrals,

$$V_{\text{int}} = \frac{(8\pi^2)^2}{2} V,$$

where the factor $1/2$ accounts for the equal division of the two-particle configurational space between left- and right-handed arrangements. Note that in the case of flexible particles, Eq. (24) may be further averaged over a representative ensemble of molecular conformations using the numerical procedure outlined in the main text (see Materials and Methods). In this study, we use for the volume V the smallest cubic box containing all possible interacting configurations of any two origami conformations.

In the context of Eqs. (23) and (24), a system of two particles with fixed inter-axis angle θ_{12} (Fig. 2A) may adopt a thermodynamically-stable right-handed configuration if their net repulsion is minimised in a right-handed arrangement — i.e., if $\overline{U}_+(\theta_{12}) < \overline{U}_-(\theta_{12})$. Conversely, $\overline{U}_+(\theta_{12}) > \overline{U}_-(\theta_{12})$ indicates a thermodynamic preference for left-handed arrangements. The relative stability of chiral two-particle assemblies is thus quantified by the chiral component of the PMF,

$$\Delta_c \overline{U}(\theta) \equiv \overline{U}_+(\theta) - \overline{U}_-(\theta) = k_b T \log \frac{\left\langle e^{-\beta U_{\text{inter}}} \right\rangle_-^{(\theta)}}{\left\langle e^{-\beta U_{\text{inter}}} \right\rangle_+^{(\theta)}}. \tag{25}$$

In the case of particles with high aspect ratios interacting through short-ranged repulsive potentials, it is easy to verify that only a small statistical fraction of the configurations sampled in Eq. (24) may display a significant interaction energy $U_{\text{inter}} > 0$, so that

$$\left\langle e^{-\beta U_{\text{inter}}} \right\rangle_\pm^{(\theta)} \longrightarrow 1 \qquad \forall \theta \in [-\pi/2, \pi/2].$$

The Taylor expansion of Eq. (25) then reads as, to leading order in $1 - \left\langle e^{-\beta U_{\text{inter}}} \right\rangle_\pm$,

$$\Delta_c \overline{U}(\theta) = k_b T \left\{ \left\langle e^{-\beta U_{\text{inter}}} \right\rangle_-^{(\theta)} - \left\langle e^{-\beta U_{\text{inter}}} \right\rangle_+^{(\theta)} \right\},$$

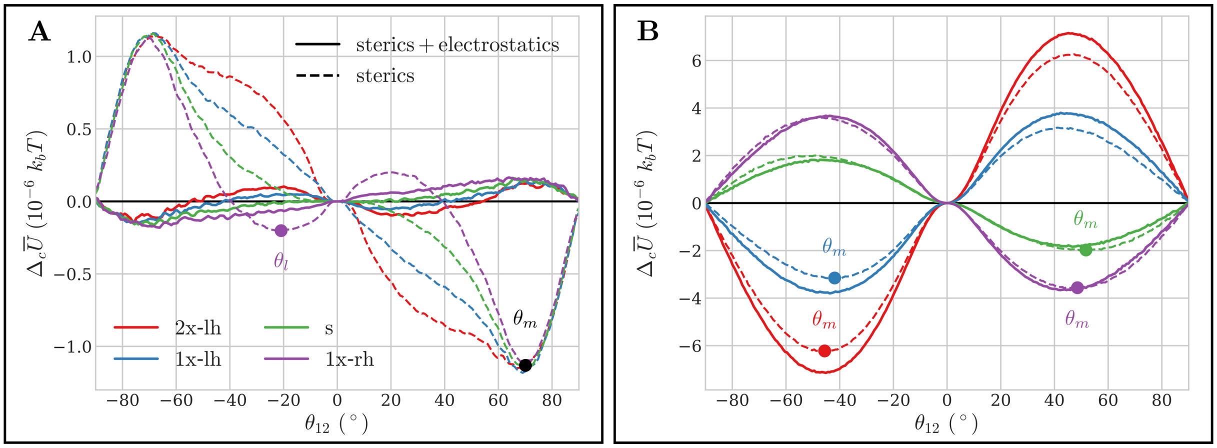

Figure S1: **Chiral two-body PMF of ground-state and thermalised origamis.** A) Chiral component of the angular PMF ($\Delta_c\overline{U}$) as a function of origami inter-axis angle (θ_{12}, see Fig. 2A) for ground-state filaments. Positive (resp. negative) values of θ_{12} denote right-handed (resp. left-handed) two-particle arrangements. Solid dots mark the locations of the curve minima, as discussed in the text, and are only displayed in the case of pure steric repulsion for clarity. B) Same as A) for thermalised origamis.

and one recovers the definition of the chiral pair excluded volume employed in Refs. (*48*) and (*26*) for systems of hard particles, up to a constant multiplicative prefactor.

It is apparent from Fig. S1 that the chiral PMFs of thermalised origamis are significantly larger in magnitude than those of their respective ground states, and are also relatively insensitive to the inclusion of electrostatic repulsion. These two observations evidence the ascendency of long-wavelength backbone deformations over local axial twist in their LChLC ordering, as the larger lengthscales associated with solenoidal writhe render the chiral assembly of thermalised filaments largely independent of the detailed nature of their much shorter-ranged repulsive interactions. The PMFs of thermalised origamis are further found to bear a unique minimum θ_m such that $\theta_m < 0$ for left-twisted filaments and $\theta_m > 0$ for their right-twisted counterparts (Fig. S1B), thus ensuring their stabilisation of iso-chiral LChLC arrangements; a thorough discussion of the quantitative link between phase handedness and chiral PMFs may be found in Ref. (*26*).

Conversely, the PMFs of ground-state filaments interacting purely through steric repulsion display a shallower minimum at large inter-axis angles ($\theta_m \simeq +70°$, Fig. S1A), corresponding to the close-approach configuration of ground-state duplexes, as the helical threads of B-DNA form a fixed angle of roughly $35°$ with respect to the normal to the double-helix axis (*27*). This large value is obviously incompatible with the local orientational order of LChLCs, but is nonetheless associated with a regime of weakly-negative values of $\Delta_c \overline{U}$ at smaller angles $\theta_{12} > 0$ in the case of the s, 1x-lh and 2x-lh origami variants — and thus leads to their formation of stable right-handed phases. However, the chiral PMF of 1x-rh filaments bears a local secondary minimum θ_l at small inter-axis angles of about $-20°$ (Fig. S1A), arising from their weak right-handed axial twist, which instead stabilises their left-handed LChLC assembly.

Finally, we report that electrostatic interactions greatly reduce the magnitude of the chiral PMFs for all ground-state filaments, indicating that the inclusion of longer-ranged repulsion results in an effective screening of their local chiral molecular surfaces — and therefore unwinds their equilibrium pitches (*32*). This conclusion is consistent with the recent results of extensive all-atom simulations of short DNA oligomers (*31*), in which the net contribution of electrostatic interactions to the chiral PMF was found to be negligible at comparable monovalent salt concentrations.

S3 Oseen-Frank twist elastic moduli and chiral strengths

We reproduce in Fig. S2 the density dependence of the Oseen-Frank twist elastic modulus K_2 and chiral strength k_t in the case of thermalised origami filaments, computed following the procedure outlined in the main text (see Materials and Methods). The orders of magnitudes of the obtained values are in very good agreement with experimental measurements performed in filamentous virus solutions (*52*), whose molecular dimensions, relative flexibility and absolute cholesteric pitches are comparable to those of the origamis (*21*). The general tendencies appar-

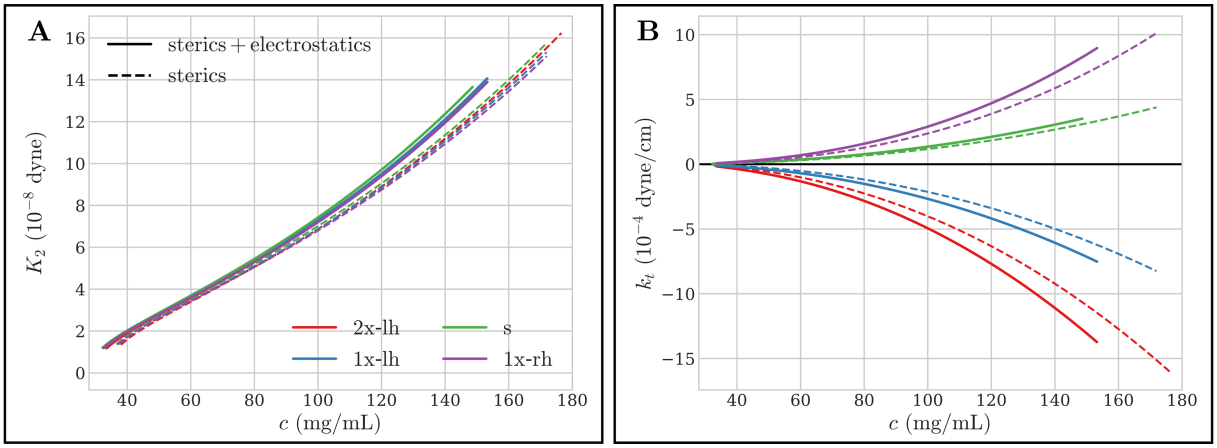

Figure S2: **Twist elastic modulus and chiral strength of thermalised origamis.** A) Oseen-Frank twist modulus (K_2) as a function of particle concentration (c) for the different thermalised origami variants. B) Same as A) for the chiral strength (k_t).

ent in Fig. S2 are also consistent with experimental results on virus assemblies, with both K_2 and k_t displaying a marked increase in magnitude with increasing particle concentration (*52*). The observed stiffening of twist curvature elasticities upon the inclusion of electrostatic repulsion (Fig. S2A) further mirrors the experimental variations of K_2 with decreasing salt concentration in such systems (*52*). The precise experimental determination of these quantities in LChLC phases of origami filaments would be desirable for the thorough investigation of these effects, and for further quantitative comparisons with the theoretical predictions of Fig. S2.

S4 Derivation of an helicity order parameter

Let us parametrise an arbitrary backbone conformation of an origami with contour length l_c by a continuous curve $\mathbf{r}(s)$, where $s \in [0, l_c]$ is the curvilinear abscissa. The local unit tangent to the curve reads as

$$\mathbf{t}(s) = \frac{d\mathbf{r}}{ds} \equiv t_{\parallel}(s)\mathbf{u} + \mathbf{t}_{\perp}(s), \tag{26}$$

with $\mathbf{u}$ the long axis of the conformation as defined in the main text (see Materials and Methods) and $\mathbf{t}_{\perp} \cdot \mathbf{u} = 0$. Due to the large bending rigidity of the filaments, we assume the transverse

fluctuations of $\mathbf{r}$ to be small,

$$\|\mathbf{t}_\perp(s)\| = \left\| \frac{d\mathbf{r}_\perp}{ds} \right\| \ll 1,$$

where we used the notation of Fig. 3A in the main text, with $\|\cdot\|$ the Euclidean norm. Thus,

$$t_\|(s) = \sqrt{1 - \|\mathbf{t}_\perp(s)\|^2} = 1 + \mathcal{O}\big(\|\mathbf{t}_\perp\|^2\big),$$

and integrating Eq. (26) yields, to leading order in $\mathbf{t}_\perp$,

$$\mathbf{r}(s) = \mathbf{r}_0 + s\mathbf{u} + \mathbf{r}_\perp(s), \tag{27}$$

where $\mathbf{r}_0 = \mathbf{r}(0) - \mathbf{r}_\perp(0)$. Consistently with the previous approximations, we further assimilate the filament long axis $\mathbf{u}$ with the normalised end-to-end separation vector,

$$\mathbf{u} \cong \frac{\mathbf{r}(l_c) - \mathbf{r}(0)}{\|\mathbf{r}(l_c) - \mathbf{r}(0)\|},$$

so that Eq. (27) imposes simple periodic boundary conditions for $\mathbf{r}_\perp$,

$$\mathbf{r}_\perp(0) = \mathbf{r}_\perp(l_c).$$

$\mathbf{r}_\perp$ may then be expressed in the form of an inverse Fourier transform,

$$\mathbf{r}_\perp(s) = \frac{1}{l_c} \sum_k \widehat{\mathbf{r}}_\perp(k) \times e^{2i\pi ks}, \tag{28}$$

with discrete wavenumbers $k = n/l_c$ for any non-zero integer n and coefficients

$$\widehat{\mathbf{r}}_\perp(k) = \int_0^{l_c} ds\, \mathbf{r}_\perp(s) \times e^{-2i\pi ks} \equiv \widehat{r}_{\perp v}(k)\mathbf{v} + \widehat{r}_{\perp w}(k)\mathbf{w}. \tag{29}$$

Let $|\cdot|$ be the complex modulus, and

$$e^{i\phi_v(k)} \equiv \frac{\widehat{r}_{\perp v}(k)}{|\widehat{r}_{\perp v}(k)|}, \tag{30}$$

$$e^{i\phi_w(k)} \equiv \frac{\widehat{r}_{\perp w}(k)}{|\widehat{r}_{\perp w}(k)|}. \tag{31}$$

Using Eqs. (27)–(31), the backbone conformation $\mathbf{r}_k$ associated with a transverse deformation mode of arbitrary wavenumber k is given by the parametric equation

$$\mathbf{r}_k(s) = s\mathbf{u} + \frac{2}{l_c}\Big\{ |\widehat{r}_{\perp v}(k)| \cos[2\pi ks + \phi_v(k)]\mathbf{v} + |\widehat{r}_{\perp w}(k)| \cos[2\pi ks + \phi_w(k)]\mathbf{w}\Big\}. \tag{32}$$

In the most general case, Eq. (32) describes an elliptical helix of axis $\mathbf{u}$ and pitch $p = 1/k$. The shape chirality associated with a deformation mode $\mathbf{r}_k$ is thus quantified by the anisotropy of its elliptical cross-section, which we now proceed to analyse. In the following, we omit some of the explicit k dependences in order to alleviate the notations when no confusion can arise. Let us denote by

$$\|\widehat{\mathbf{r}}_\perp\| \equiv \sqrt{\widehat{\mathbf{r}}_\perp \cdot \widehat{\mathbf{r}}_\perp^*} = \sqrt{|\widehat{r}_{\perp v}|^2 + |\widehat{r}_{\perp w}|^2} \tag{33}$$

the Euclidean modulus of $\widehat{\mathbf{r}}_\perp$, and define

$$\theta \equiv \arccos \frac{|\widehat{r}_{\perp v}|}{\|\widehat{\mathbf{r}}_\perp\|}, \tag{34}$$

$$A \equiv \frac{2\|\widehat{\mathbf{r}}_\perp\|}{l_c}. \tag{35}$$

Using Eqs. (32)–(35), the transverse components of $\mathbf{r}_k$ may be rewritten as

$$r_{kv}(s) \equiv \mathbf{r}_k(s) \cdot \mathbf{v} = A\cos\theta \times \cos(\omega s + \phi_v), \tag{36}$$

$$r_{kw}(s) \equiv \mathbf{r}_k(s) \cdot \mathbf{w} = A\sin\theta \times \cos(\omega s + \phi_w), \tag{37}$$

with $\omega \equiv 2\pi k$. Eq. (37) then yields

$$\frac{r_{kw}(s)}{A\sin\theta} = \cos(\omega s + \phi_v)\cos\phi + \sin(\omega s + \phi_v)\sin\phi,$$

where

$$\phi \equiv \phi_v - \phi_w. \tag{38}$$

Thus, using Eq. (36),

$$\frac{r_{kw}(s)}{A\sin\theta} - \frac{r_{kv}(s)}{A\cos\theta}\cos\phi = \sin(\omega s + \phi_v)\sin\phi, \tag{39}$$

and Eq. (36) immediately yields the further relation

$$\sin(\omega s + \phi_v) = \pm\sqrt{1 - \left\{\frac{r_{kv}(s)}{A\cos\theta}\right\}^2}. \tag{40}$$

Plugging Eq. (40) into Eq. (39) leads to a quadratic equation for r_{kv} and r_{kw},

$$\left(\frac{r_{kv}}{A\cos\theta}\right)^2 + \left(\frac{r_{kw}}{A\sin\theta}\right)^2 - 2\cos\phi\frac{r_{kv}r_{kw}}{A^2\cos\theta\sin\theta} = \sin^2\phi. \tag{41}$$

Denoting by $\mathbf{r}_{k\perp}$ the total transverse component of $\mathbf{r}_k$,

$$\mathbf{r}_{k\perp}(s) \equiv r_{kv}(s)\mathbf{v} + r_{kw}(s)\mathbf{w},$$

Eq. (41) may be recast in the compact form

$$\mathbf{r}_{k\perp}^{\mathsf{T}} \cdot \mathcal{Q} \cdot \mathbf{r}_{k\perp} = 1,$$

with $\mathbf{r}_{k\perp}^{\mathsf{T}}$ the matrix transpose of $\mathbf{r}_{k\perp}$ and $\mathcal{Q}$ the matrix representation of the quadratic form in Eq. (41),

$$\mathcal{Q} = \frac{1}{\sin^2\phi}\begin{bmatrix} \dfrac{1}{(A\cos\theta)^2} & -\dfrac{\cos\phi}{A^2\cos\theta\sin\theta} \\ -\dfrac{\cos\phi}{A^2\cos\theta\sin\theta} & \dfrac{1}{(A\sin\theta)^2} \end{bmatrix}.$$

The respective lengths $r_\pm$ of the semi-major and semi-minor elliptical axes are then related to the respective largest and smallest eigenvalues $\lambda_\pm$ of $\mathcal{Q}$ through (*53*)

$$r_\pm = 1/\sqrt{\lambda_\mp},$$

which yields, after rearrangements,

$$r_\pm = A\sqrt{\frac{1 \pm \sqrt{1 - \sin^2\phi\sin^2 2\theta}}{2}}. \tag{42}$$

Interestingly, Eq. (42) bears a strong resemblance to the Jones vector parametrisation of the polarisation ellipse in classical electrodynamics (*54*), which stems from the similarity between

Eq. (32) and the field equation of a polarised electromagnetic wave propagating along the direction $\mathbf{u}$.

Let us define

$$\mathcal{H} \equiv \sin\phi \sin 2\theta. \tag{43}$$

An explicit expression for $\mathcal{H}$ in terms of the Fourier components $\widehat{\mathbf{r}}_\perp(k)$ may be obtained by substituting Eqs. (33) and (34) for θ,

$$\sin 2\theta = 2\cos\theta\sin\theta = \frac{2\,|\widehat{r}_{\perp v}|\,|\widehat{r}_{\perp w}|}{|\widehat{r}_{\perp v}|^2 + |\widehat{r}_{\perp w}|^2},$$

and substituting Eqs. (38), (30) and (31) for ϕ,

$$e^{i\phi} = e^{i\phi_v}e^{-i\phi_w} = \frac{\widehat{r}_{\perp v} \times \widehat{r}^*_{\perp w}}{|\widehat{r}_{\perp v}|\,|\widehat{r}_{\perp w}|}.$$

Eq. (43) may thus be rewritten in the form

$$\mathcal{H} = 2 \times \frac{\Im\{\widehat{r}_{\perp v} \times \widehat{r}^*_{\perp w}\}}{|\widehat{r}_{\perp v}|^2 + |\widehat{r}_{\perp w}|^2} = 2 \times \frac{\Im\{\widehat{c}_{vw}\}}{\widehat{c}_{vv} + \widehat{c}_{ww}}, \tag{44}$$

and one recovers the definition of Eq. 4 in the main text, with $\widehat{c}_{vw}(k)$ the Fourier components of the cross-correlation function of $r_{\perp v}$ and $r_{\perp w}$ as given by the convolution theorem,

$$\widehat{c}_{vw}(k) = \widehat{r}_{\perp v}(k) \times \widehat{r}^*_{\perp w}(k).$$

Using Eqs. (42) and (43), the transverse eccentricity of the elliptical cross-section reads as

$$e^2 \equiv \frac{r_+^2 - r_-^2}{r_+^2} = \frac{2\sqrt{1-\mathcal{H}^2}}{1+\sqrt{1-\mathcal{H}^2}}.$$

A necessary and sufficient condition for the deformation mode $\mathbf{r}_k$ to describe an ideal circular helix is given by

$$e(k) = 0 \iff \mathcal{H}(k) = \pm 1.$$

Conversely,

$$e(k) = 1 \iff \mathcal{H}(k) = 0$$

describes the degenerate case in which the elliptical cross-section collapses to a flat line segment, leading to an achiral deformation mode. The mean solenoidal radius r_m of an arbitrary deformation mode may finally be obtained in the compact form

$$r_m \equiv \sqrt{r_+ \times r_-} = A\sqrt{\frac{|\mathcal{H}|}{2}}.$$

The magnitude of $\mathcal{H}(k)$ may thus be understood as a measure of the degree of circular helicity of the deformation mode $\mathbf{r}_k$. The link between the sign of $\mathcal{H}(k)$ and the corresponding helical handedness may be elucidated by considering the case of an ideal circular helical conformation of axis $\mathbf{u}$, radius $r_h > 0$ and inverse pitch $q = 1/p_h$. The general parametric equation of such a conformation reads as, in the limit of weak helical curvature ($qr_h \ll 1$),

$$\mathbf{r}_q^h(s) = s\mathbf{u} + r_h \cos(2\pi qs + \phi_h)\mathbf{v} + r_h \sin(2\pi qs + \phi_h)\mathbf{w}, \tag{45}$$

with $\phi_h \in [0, 2\pi]$. In the convention of Eq. (45), the handedness of the helix is quantified by the sign of q, with $q > 0$ (resp. $q < 0$) corresponding to a right-handed (resp. left-handed) helicity. Using the previous notations, the Fourier components of the transverse vector $\mathbf{r}_{q\perp}^h$ associated with Eq. (45) read as

$$\widehat{\mathbf{r}}_{q\perp}^h(k) = \begin{cases} l_c \times \dfrac{r_h e^{\pm i\phi_h}}{2}\big(\mathbf{v} \pm e^{-i\pi/2}\mathbf{w}\big) & \text{if} \quad k \ = \pm q \\ \mathbf{0} & \text{if} \ |k| \neq |q| \end{cases}.$$

In this case, for any wavenumber $k > 0$, Eq. (44) reduces to

$$\mathcal{H}(k) = \delta_{k,|q|} \times \operatorname{sgn} q,$$

and it is easy to check that Eqs. (42), (33) and (35) yield

$$r_+(k) = r_-(k) = \delta_{k,|q|} \times r_h,$$

with δ the Kronecker delta and sgn the sign function. Therefore, the handedness of a deformation mode with arbitrary wavenumber $k > 0$ may be determined by the sign of $\mathcal{H}(k)$, with $\mathcal{H}(k) > 0$ and $\mathcal{H}(k) < 0$ respectively describing a right- and left-handed helicity.

S5 Fluctuation spectrum from the equipartition theorem

Using the notations of Section S4, the enthalpic penalty associated with the bending response of a single origami to thermal fluctuations reads as, in the case of weak curvature deformations,

$$\Delta H_{\rm bend} = \frac{\mathcal{K}}{2} \int_0^{l_c} ds \left\| \frac{d^2 \mathbf{r}_\perp}{ds^2} \right\|^2, \tag{46}$$

where the bending modulus $\mathcal{K}$ is related to the origami persistence length l_p through

$$\mathcal{K} = l_p k_b T. \tag{47}$$

Substituting Eqs. (28) and (29) for $\mathbf{r}_\perp$ in Eq. (46) yields

$$\Delta H_{\rm bend} = \frac{\mathcal{K}}{2 l_c} \times \sum_k (2\pi k)^4 \Big\{ \, |\widehat{r}_{\perp v}(k)|^2 + |\widehat{r}_{\perp w}(k)|^2 \, \Big\}. \tag{48}$$

Assimilating the different transverse deformation modes in Eq. (48) to decoupled degrees of freedom, the equipartition theorem imposes for $\widehat{r}_{\perp v}$ and $\widehat{r}_{\perp w}$

$$\big\langle \, |\widehat{r}_{\perp v}(k)|^2 \, \big\rangle = \big\langle \, |\widehat{r}_{\perp w}(k)|^2 \, \big\rangle = \frac{k_b T l_c}{\mathcal{K}} \times \frac{1}{(2\pi k)^4}.$$

Thus, using Eqs. (33) and (47),

$$\big\langle \|\widehat{\mathbf{r}}_\perp(k)\|^2 \big\rangle = \frac{l_c}{l_p} \times \frac{1}{8\pi^4 k^4}, \tag{49}$$

valid in the limit of long-wavelength fluctuations ($k \to 0$).

S6 Twist-writhe conversion and helical fluctuations

Let us consider a long origami filament whose extremities are firmly clamped to impose the parallel alignment of its backbone end tangents,

$$\left.\frac{d\mathbf{r}}{ds}\right|_{s=0} = \left.\frac{d\mathbf{r}}{ds}\right|_{s=l_c} \equiv \mathbf{t}_0. \tag{50}$$

The origami backbone curve $\mathbf{r}$ is defined as

$$\mathbf{r} \equiv \frac{1}{6} \sum_{i=1}^{6} \mathbf{r}_i,$$

where the continuous centerline $\mathbf{r}_i(s_i)$ of the i-th constituent DNA duplex is obtained by contour interpolation of the center-of-mass positions of its bonded nucleotides (see Materials and Methods). For simplicity, we neglect the effects of duplex splaying at the origami ends, and thus assume Eq. (50) to hold at each of the center curve extremities,

$$\left.\frac{d\mathbf{r}_i}{ds_i}\right|_{s_i=0} = \left.\frac{d\mathbf{r}_i}{ds_i}\right|_{s_i=l_i} = \mathbf{t}_0.$$

We further restrict our study to the regime of weak bending deformations of the duplex centerlines about the straight backbone conformation of the origami ground state, and neglect potential fluctuations in their respective contour lengths l_i.

Under these assumptions, the formulation of the Călugăreanu-Fuller-White theorem extended to the treatment of open curves (*41*) states that the linking number Lk_i of each individual duplex may be decomposed into twist and writhe contributions,

$$\mathrm{Lk}_i = \mathrm{Tw}_i + \mathrm{Wr}_i. \tag{51}$$

In this context, Lk_i represents the (signed) number of net right-handed turns per unit contour length by which the two strands of the duplex wind around $\mathbf{t}_0$ (*41*). These turns may result in both a local twist of the strands about their common centerline $\mathbf{r}_i$, as quantified by the twist density Tw_i, and/or in a global supercoiling of the centerline itself, as measured by the writhe integral Wr_i. It should be noted that the linking number Lk_i is generally not a topological invariant in the case of non-circular DNA fragments. Within the origami filament architecture, Lk_i is initially constrained by the designed locations of the inter-helical crossovers, but may partially relax towards its preferred unhindered value Lk_0 — thus inducing global axial twist in the origami ground state (*22*).

Within ground-state B-DNA, the relaxed linking number Lk_0 is entirely absorbed in the form of twist strain,

$$\mathrm{Lk}_0 = \mathrm{Tw}_0 \simeq \frac{1}{10.5}\,\mathrm{bp}^{-1}. \tag{52}$$

The axial twist handedness of an origami filament comprised of duplexes with linking number Lk_i is therefore determined by the sign of $\Delta\mathrm{Lk}_i \equiv \mathrm{Lk}_i - \mathrm{Lk}_0$, with $\Delta\mathrm{Lk}_i > 0$ ($\Delta\mathrm{Lk}_i < 0$) respectively denoting a residual over-winding (under-winding) of the duplexes, associated with a global left-handed (right-handed) compensatory twist of the origami. The total elastic energy of a constituent duplex, as defined by an arbitrary centerline curve $\mathbf{r}_i$ and uniform twist density Tw_i, may be obtained as a straightforward generalisation of Eq. (46) (*55*),

$$\Delta H_i = \frac{1}{2}\int_0^{l_i} ds_i \left\{ \mathcal{K}_i \left\| \frac{d^2\mathbf{r}_i}{ds_i^2} \right\|^2 + 4\pi^2 C_i \Big(\mathrm{Tw}_i - \mathrm{Tw}_0\Big)^2 \right\},$$

with $\mathcal{K}_i$ and C_i the respective effective bending and twisting moduli of B-DNA within the origami structure. Eqs. (51) and (52) immediately yield

$$\Delta H_i = \frac{\mathcal{K}_i}{2}\int_0^{l_i} ds_i \left\| \frac{d^2\mathbf{r}_i}{ds_i^2} \right\|^2 + 2\pi^2 C_i l_i \Big(\Delta\mathrm{Lk}_i - \mathrm{Wr}_i\Big)^2. \tag{53}$$

It is apparent that the twist elastic contribution in Eq. (53) is minimised by conformations in which $\Delta\mathrm{Lk}_i$ and Wr_i bear equal sign and magnitude, leading to a favoured positive (right-handed) supercoiling in the case of left-twisted origamis ($\Delta\mathrm{Lk}_i > 0$, $\mathrm{Wr}_i > 0$), and negative (left-handed) supercoiling for their right-handed counterparts ($\Delta\mathrm{Lk}_i < 0$, $\mathrm{Wr}_i < 0$). However, this twist relaxation mechanism is hindered by the high penalty in bending energy arising from the finite curvature of the resulting solenoidal centerline deformations. The competition of these two effects, acting constructively on each duplex within the origami structures, leads to the weak anti-chiral backbone fluctuations underpinning their LChLC assembly.

REFERENCES AND NOTES

1. S. C. Glotzer, M. J. Solomon, Anisotropy of building blocks and their assembly into complex structures. *Nat. Mater.* **6**, 557–562 (2007).

2. A. Stein, B. E. Wilson, S. G. Rudisill, Design and functionality of colloidal-crystal-templated materials—Chemical applications of inverse opals. *Chem. Soc. Rev.* **42**, 2763–2803 (2013).

3. M. Beija, R. Salvayre, N. Lauth-de Viguerie, J.-D. Marty, Colloidal systems for drug delivery: From design to therapy. *Trends Biotechnol.* **30**, 485–496 (2012).

4. E. Elacqua, X. Zheng, C. Shillingford, M. Liu, M. Weck, Molecular recognition in the colloidal world. *Acc. Chem. Res.* **50**, 2756–2766 (2017).

5. Y. Wang, J. Xu, Y. Wang, H. Chen, Emerging chirality in nanoscience. *Chem. Soc. Rev.* **42**, 2930–2962 (2013).

6. M. Hentschel, M. Schäferling, X. Duan, H. Giessen, N. Liu, Chiral plasmonics. *Sci. Adv.* **3**, e1602735 (2017).

7. J. D. Bernal, *The Origin of Life* (World Publishing Company, 1967).

8. E. Yashima, N. Ousaka, D. Taura, K. Shimomura, T. Ikai, K. Maeda, Supramolecular helical systems: Helical assemblies of small molecules, foldamers, and polymers with chiral amplification and their functions. *Chem. Rev.* **116**, 13752–13990 (2016).

9. F. Reinitzer, Beiträge zur Kenntniss des Cholesterins. *Monatsh. Chem.* **9**, 421–441 (1888).

10. C. B. Stanley, H. Hong, H. H. Strey, DNA cholesteric pitch as a function of density and ionic strength. *Biophys. J.* **89**, 2552–2557 (2005).

11. E. Grelet, S. Fraden, What is the origin of chirality in the cholesteric phase of virus suspensions?. *Phys. Rev. Lett.* **90**, 198302 (2003).

12. P. De Sa Peixoto, A. Deniset-Besseau, M.-C. Schanne-Klein, G. Mosser, Quantitative assessment of collagen I liquid crystal organizations: role of ionic force and acidic solvent, and evidence of new phases. *Soft Matter* **7**, 11203–11210 (2011).

13. M. Schwartz, G. Lenzini, Y. Geng, P.B. Rønne, P.Y.A. Ryan, J.P.F. Lagerwall, Cholesteric liquid crystal shells as enabling material for information-rich design and architecture. *Adv. Mater.* **30**, 1707382 (2018).

14. C. De Michele, G. Zanchetta, T. Bellini, E. Frezza, A. Ferrarini, Hierarchical propagation of chirality through reversible polymerization: The cholesteric phase of DNA oligomers. *ACS Macro Lett.* **5**, 208–212 (2016).

15. H. H. Wensink, G. Jackson, Cholesteric order in systems of helical Yukawa rods. *J. Phys. Condens. Matter* **23**, 194107 (2011).

16. E. Frezza, A. Ferrarini, H. B. Kolli, A. Giacometti, G. Cinacchi, Left or right cholesterics? A matter of helix handedness and curliness. *Phys. Chem. Chem. Phys.* **16**, 16225–16232 (2014).

17. S. Dussi, M. Dijkstra, Entropy-driven formation of chiral nematic phases by computer simulations. *Nat. Commun.* **7**, 11175 (2016).

18. J. P. F. Lagerwall, C. Schütz, M. Salajkova, J.H. Noh, J. Hyun Park, G. Scalia, L. Bergström, Cellulose nanocrystal-based materials: From liquid crystal self-assembly and glass formation to multifunctional thin films. *NPG Asia Mater.* **6**, e80 (2014).

19. L. Wang, A. M. Urbas, Q. Li, Nature-inspired emerging chiral liquid crystal nanostructures: From molecular self-assembly to DNA mesophase and nanocolloids. *Adv. Mater.* **2018**, 1801335 (2018).

20. M. Mitov, Cholesteric liquid crystals in living matter. *Soft Matter* **13**, 4176–4209.

21. M. Siavashpouri, C. H. Wachauf, M. J. Zakhary, F. Praetorius, H. Dietz, Z. Dogic, 856, Molecular engineering of chiral colloidal liquid crystals using DNA origami. *Nat. Mater.* **16**, 849–856 (2017).

22. H. Dietz, S. M. Douglas, W. M. Shih, Folding DNA into twisted and curved nanoscale shapes. *Science* **325**, 725–730 (2009).

23. D.-N. Kim, F. Kilchherr, H. Dietz, M. Bathe, 2868, Quantitative prediction of 3D solution shape and flexibility of nucleic acid nanostructures. *Nucleic Acids Res.* **40**, 2862–2868 (2012).

24. J. P. Straley, Theory of piezoelectricity in nematic liquid crystals, and of the cholesteric ordering. *Phys. Rev. A* **14**, 1835–1841 (1976).

25. M. M. C. Tortora, J. P. K. Doye, Perturbative density functional methods for cholesteric liquid crystals. *J. Chem. Phys.* **146**, 184504 (2017).

26. M. M. C. Tortora, J. P. K. Doye, Hierarchical bounding structures for efficient virial computations: Towards a realistic molecular description of cholesterics. *J. Chem. Phys.* **147**, 224504 (2017).

27. F. Tombolato, A. Ferrarini, From the double-stranded helix to the chiral nematic phase of B-DNA: A molecular model. *J. Chem. Phys.* **122**, 054908 (2005).

28. B. E. K. Snodin, F. Randisi, M. Mosayebi, P. Šulc, J. S. Schreck, F. Romano, T. E. Ouldridge, R. Tsukanov, E. Nir, A. A. Louis, J. P. K. Doye, Introducing improved structural

properties and salt dependence into a coarse-grained model of DNA. *J. Chem. Phys.* **142**, 234901 (2015).

29. A. A. Kornyshev, S. Leikin, S. V. Malinin, Chiral electrostatic interaction and cholesteric liquid crystals of DNA. *Eur. Phys. J. E* **7**, 83–93 (2002).

30. A. G. Cherstvy, DNA cholesteric phases: The role of DNA molecular chirality and DNA−DNA electrostatic interactions. *J. Phys. Chem. B* **142**, 12585 (2008).

31. R. Cortini, X. Cheng, J. C. Smith, The tilt-dependent potential of mean force of a pair of DNA oligomers from all-atom molecular dynamics simulations. *J. Phys. Condens. Matter* **29**, 084002 (2017).

32. J. Araki, S. Kuga, Effect of trace electrolyte on liquid crystal type of cellulose microcrystals. *Langmuir* **17**, 4493–4496 (2001).

33. M. M. C. Tortora, J. P. K. Doye, Incorporating particle flexibility in a density functional description of nematics and cholesterics. *Mol. Phys.* **116**, 2773–2791 (2018).

34. H. Fynewever, A. Yethiraj, Phase behavior of semiflexible tangent hard sphere chains. *J. Chem. Phys.* **108**, 1636–1644 (1998).

35. D. Schiffels, T. Liedl, D. K. Fygenson, Nanoscale structure and microscale stiffness of DNA nanotubes. *ACS Nano* **7**, 6700–6710 (2013).

36. F. Tombolato, A. Ferrarini, E. Grelet, Chiral nematic phase of suspensions of rodlike viruses: Left-handed phase helicity from a right-handed molecular helix. *Phys. Rev. Lett.* **96**, 258302 (2006).

37. T. Odijk, Pitch of a polymer cholesteric. *J. Phys. Chem.* **91**, 6060–6062 (1987).

38. D. M. Hall, I. R. Bruss, J. R. Barone, G. M. Grason, Morphology selection via geometric frustration in chiral filament bundles. *Nat. Mater.* **15**, 727–732 (2016).

39. J. Adamcik, J.M. Jung, J. Flakowski, P. de Los Rios, G. Dietler, R. Mezzenga, Understanding amyloid aggregation by statistical analysis of atomic force microscopy images. *Nat. Nanotechnol.* **5**, 423–428 (2010).

40. M. Sayar, B. Avşaroğlu, A. Kabakçıoğlu, Twist-writhe partitioning in a coarse-grained DNA minicircle model. *Phys. Rev. E* **81**, 041916 (2010).

41. M. A. Berger, C. Prior, The writhe of open and closed curves. *J. Phys. A Math. Gen.* **39**, 8321–8348 (2006).

42. A. Savitzky, M. J. E. Golay, Smoothing and differentiation of data by simplified least squares procedures. *Anal. Chem.* **36**, 1627–1639 (1964).

43. P.-G. de Gennes, J. Prost, *The Physics of Liquid Crystals* (Clarendon Press, 1993).

44. J. Herzfeld, A. E. Berger, J. W. Wingate, A highly convergent algorithm for computing the orientation distribution functions of rodlike particles. *Macromolecules* **17**, 1718–1723 (1984).

45. D. Chandler, J. D. McCoy, S. J. Singer, Density functional theory of nonuniform polyatomic systems. I. General formulation. *J. Chem. Phys.* **85**, 5971–5976 (1986).

46. T. van Westen, T. J. Vlugt, J. Gross, An analytical approximation for the orientation-dependent excluded volume of tangent hard sphere chains of arbitrary chain length and flexibility. *J. Chem. Phys.* **137**, 044906 (2012).

47. K. M. Jaffer, S. B. Opps, D. E. Sullivan, B. G. Nickel, L. Mederos, The nematic-isotropic phase transition in semiflexible fused hard-sphere chain fluids. *J. Chem. Phys.* **114**, 3314–3324 (2001).

48. S. Dussi, S. Belli, R. van Roij, M. Dijkstra, Cholesterics of colloidal helices: Predicting the macroscopic pitch from the particle shape and thermodynamic state. *J. Chem. Phys.* **142**, 074905 (2015).

49. R. B. Meyer, *Polymer Liquid Crystals*, A. Ciferri, W. Krigbaum, R. B. Meyer, Eds. (Academic Press, 1982), chap. 6, pp. 133–163.

50. A. Poniewierski, J. Stecki, Statistical theory of the elastic constants of nematic liquid crystals. *Mol. Phys.* **38**, 1931–1940 (1979).

51. H. H. Wensink, Spontaneous sense inversion in helical mesophases. *Europhys. Lett.* **107**, 36001 (2014).

52. Z. Dogic, S. Fraden, Cholesteric phase in virus suspensions. *Langmuir* **16**, 7820–7824 (2000).

53. G. Salmon, *A Treatise on Conic Sections* (AMS Chelsea Publishing, ed. 6, 2000).

54. R. C. Jones, A new calculus for the treatment of optical systems. I. Description and discussion of the calculus. *J. Opt. Soc. Am.* **31**, 488–493 (1941).

55. J. F. Marko, E. D. Siggia, Statistical mechanics of supercoiled DNA. *Phys. Rev. E* **52**, 2912–2938 (1995).
